# Respiratory Effects of Continuous Positive Airway Pressure Administered during Recovery from General Anesthesia in Brachycephalic Dogs

**DOI:** 10.3390/vetsci11020075

**Published:** 2024-02-06

**Authors:** Caterina Vicenti, Pablo E. Otero, Angela Briganti, Vincenzo Rondelli, Marzia Stabile, Claudia Piemontese, Antonio Crovace, Luca Lacitignola, Francesco Staffieri

**Affiliations:** 1 Section of Veterinary Clinics and Animal Production, Department of Precision and Regenerative Medicine and Ionian Area (DiMePre-J), University of Bari, 70010 Valenzano, Italy; caterina.vicenti@uniba.it (C.V.); potero@fvet.uba.ar (P.E.O.);; 2 Department of Anesthesiology and Pain Management, Facultad de Ciencias Veterinarias, Universidad de Buenos Aires, Buenos Aires C1427CWN, Argentina; 3 Department of Veterinary Sciences, Veterinary Teaching Hospital “Mario Modenato”, University of Pisa, 56122 Pisa, Italy; 4 Centro Veterinario Gregorio VII—BluVet, 00165 Rome, Italy; vincenzorondelli@gregoriovii.com

**Keywords:** brachycephalic, dog, CPAP, oxygenation, anesthesia

## Abstract

**Simple Summary:**

Brachycephalic dogs are more prone to develop upper airway respiratory complications in the postoperative period. The application of pre- and postoperative continuous positive airway pressure (CPAP) therapy has been suggested in human patients with obstructive sleep apnea to enhance oxygenation, diminish airway swelling, reduce the work of breathing, and promote recovery. Brachycephalic dogs undergoing various surgical procedures were included in this study and assigned to receive either standard oxygen supplementation (NO-CPAP group) or oxygen supplementation combined with CPAP (CPAP group). The CPAP group showed significant improvements in respiratory function compared with the NO-CPAP group (*p* < 0.001). The incidence of reintubation and helmet intolerance was higher in the NO-CPAP group (18% and 15.6%, respectively) than in the CPAP group (0%). This study highlights the potential benefits of incorporating CPAP, delivered through a pediatric helmet, in the perioperative management of brachycephalic dogs.

**Abstract:**

This study aimed to evaluate the benefits of applying 5 cmH_2_O of CPAP using a pediatric helmet during the recovery phase from general anesthesia in brachycephalic dogs. Brachycephalic dogs undergoing various surgical procedures were included in this study, and a total of 64 subjects were randomly assigned to receive either standard oxygen supplementation (NO-CPAP group) or oxygen supplementation combined with CPAP (CPAP group). This study evaluated arterial blood pH, blood gas partial pressures of O_2_ and CO_2_, arterial blood O_2_ saturation, and related parameters during recovery. The dogs were monitored, and helmet tolerance was assessed using predefined criteria. Of the initially assessed 69 dogs, 64 were enrolled: 32 in the CPAP group and 32 in the NO-CPAP group. Fifteen dogs in the NO-CPAP group were excluded based on predetermined criteria. The CPAP group showed significant improvements in PaO_2_, PaO_2_/FiO_2_, P(A-a)O_2_, F-Shunt, and respiratory rate compared with the NO-CPAP group (*p* < 0.001). The incidence of reintubation and helmet intolerance was higher in the NO-CPAP group (18% and 15.6%, respectively) than in the CPAP group (0%). This study highlights the potential benefits of incorporating CPAP, delivered through a pediatric helmet, in the perioperative management of brachycephalic dogs.

## 1. Introduction

Brachycephalic airway obstructive syndrome (BOAS) is a condition that affects dogs with short noses and can result in severe respiratory difficulties [1]. Dogs affected by BAOS exhibit a range of clinical symptoms, such as noisy or stertorous breathing, pronounced snoring, coughing, gagging, episodes of syncope, occasional collapses, and difficulties in eating [2]. Due to their peculiar anatomical characteristics, brachycephalic dogs are more prone to developing upper airway respiratory complications in the postoperative period. They may experience upper airway obstruction, resulting in an increased work of breathing and potential lower arterial partial pressure of oxygen and hypercapnia [3]. Consequently, ventilatory support may be required to protect the airway and control respiratory activity [4]. For these reasons, the recovery phase from general anesthesia emerges as a pivotal component in the perioperative management of brachycephalic dog breeds. Strategic planning of the recovery period is imperative, considering factors such as the placement of a temporary tracheostomy tube, the administration of corticosteroids, and the utilization of sedation and nebulization [5,6].

Ideally, after extubation, brachycephalic dogs should be placed in sternal recumbency with their head elevated, neck extended, and tongue rostrally drawn out of the mouth. This positioning is crucial for maintaining an open airway during the recovery phase [4]. Monitoring postoperative lung function involves using arterial blood gas measurements (especially PaO_2_), performing pulse oximetry (SpO_2_), and assessing the dog’s respiratory rate and effort [7,8,9]. Supplemental oxygen is often administered through flow-by oxygen, placement in an oxygen cage, or nasal oxygen lines [10,11,12]. In severe cases where dyspnea remains unresponsive to supplemental oxygen, dogs may need to be anesthetized and reintubated [8].

The application of pre- and postoperative continuous positive airway pressure (CPAP) therapy has been suggested in human patients with obstructive sleep apnea to enhance oxygenation, diminish airway swelling, reduce the work of breathing, and promote recovery [13].

CPAP therapy, a noninvasive ventilation mode, maintains positive airway pressure during spontaneous breathing without needing inspiratory support [14]. Its noninvasive nature eliminates the need for endotracheal intubation and allows delivery through diverse interfaces: including the Boussignac system connected to a veterinary conical face mask [15] and a helmet equipped with a CPAP valve [14].

Studies have demonstrated that, as in humans, CPAP improves ventilation in dogs and cats, elevates arterial oxygen concentration, decreases carbon dioxide levels and respiratory rate, expands upper airway diameters, reduces airway resistance and the work of breathing, and enhances functional residual capacity [14,16,17]. Furthermore, the CPAP strategy may address pulmonary atelectasis by directly recruiting alveoli, thereby lowering physiological dead space [9].

Recent research indicates that in dogs recovering from general anesthesia with impaired pulmonary gas exchange after extubation (i.e., SpO_2_ < 95%), normoxemia is more efficiently and rapidly restored through the application of CPAP with the helmet, as opposed to oxygen therapy [9]. Despite the evident efficacy of CPAP in enhancing oxygenation in animals, its utilization with the helmet during the recovery period from general anesthesia in brachycephalic breeds has yet to be investigated.

The main objective of this study was to evaluate the benefits of administering CPAP using a pediatric helmet equipped with a positive end-expiratory pressure (PEEP) valve during the recovery phase from general anesthesia in brachycephalic dogs. The hypothesis proposed that applying 5 cmH_2_O of CPAP via the helmet for 30 min after extubation can improve ventilatory function, increasing blood oxygenation. To test this hypothesis, a comparative analysis was conducted between patients receiving 30 min of standard oxygen supplementation and those receiving oxygen supplementation combined with 5 cmH_2_O of CPAP during a similar timeframe. This study aimed to evaluate specific outcomes, including arterial blood pH (pH) and blood gas partial pressures of O_2_ and CO_2_ (PaO_2_, PaCO_2_; mmHg), arterial blood O_2_ saturation (SaO_2_), and surrogate calculations such as the PaO_2_ to FiO_2_ ratio (PaO_2_/FiO_2_) and the estimated percentage of intrapulmonary shunt (F-Shunt), during the recovery period in brachycephalic dogs instrumented with the helmet. A comparison was made between those in the continuous positive airway pressure group (CPAP group) and those without continuous positive airway pressure (NO-CPAP group). Additionally, helmet tolerance was evaluated using both strategies.

## 2. Materials and Methods

This study was approved by the Ethical Committee of the Clinical and Zootechnical Studies in Animals of the Department of Emergency and Organ Transplantation of the University of Bari, Italy (n. 04/2015 D.E.T.O.), and it was conducted in three centers: University of Bari (Bari, Italy), University of Pisa (Pisa, Italy), and Veterinary Institute of Novara (Novara, Italy). This article is reported in accordance with the Consolidated Standard of Reporting Trials (CONSORT) Statement for the reporting of randomized controlled trials [18].

### 2.1. Animals

After the written owner consent, this prospective, randomized, multicenter clinical study, conducted from 2015 to 2016, included brachycephalic dogs with BOAS presented for surgical procedures related to or unrelated to the airways. Exclusion criteria were duration of anesthesia shorter than 60 min, impossibility to collect the data required (e.g., blood gas), severe intolerance to the helmet, and severe arterial blood desaturation (SpO_2_ < 85%), which required reintubation. Allocation concealment was maintained using opaque envelopes, and random assignment was conducted utilizing a sequence generator available at http://www.random.org/ (accessed on 1 January 2015).

All dogs were undergoing physical and laboratory investigation, and patients affected by any other major diseases (except BAOS) were excluded from this study. Dogs of body mass less than 5 kg and pregnant females were excluded from this study. Solid food was withheld for 6 h and water for 2 h.

### 2.2. Anesthetic Management

Dogs were admitted to the clinic two hours before general anesthesia and individually housed in separate cages. Following acclimation, all dogs intramuscularly (IM) received methadone (0.3 mg kg^−1^; Semfortan, Dechra, Turin, Italy) as premedication (PREM). After aseptic preparation, a catheter was inserted into a cephalic vein and into a dorsal pedal artery to administer fluids and drugs and to collect arterial blood samples for blood gas analysis (I-stat 1, CG8+ Cartridge, Abbott, Horwich, UK), respectively. If the arterial catheter could not be placed or did not remain patent until the end of the trial, arterial blood was obtained through percutaneous puncture of an accessible artery. General anesthesia was induced with intravenous (IV) propofol (Proposure 10 mg mL^−1^; Merial, Mantua, Italy) administered to effect after at least two minutes of preoxygenation via a facemask. The trachea was intubated with an appropriately sized cuffed endotracheal tube and connected to a circular breathing system. Anesthesia was maintained with isoflurane (end-tidal isoflurane percentage (EtISO) between 1.1 and 1.4%; [18]) in a mixture of oxygen (FiO_2_ > 0.8). A constant rate IV infusion of fentanyl (5–15 µg kg^−1^ h^−1^; Fentadon, Dechra, Turin, Italy) was intraoperatively given when necessary. All animals received lactated Ringer’s solution (Ringer Lattato, B. Braun, Milan, Italy) at 3 mL kg^−1^ h^−1^.

During anesthesia, dogs were ventilated with a volume-controlled ventilation mode using a VT of 12 mL kg^−1^ with an inspiratory to expiratory ratio of 1:2, 25% end-inspiratory pause, and 0 cmH_2_O PEEP. The respiratory rate (RR) was titrated based on the response of the dogs, considering a different target of end-tidal carbon dioxide (EtCO_2_). The initial EtCO_2_ range was 40–45 mmHg; if the RR needed to ensure this range was higher than 19 b/min, the target was modified to 45–55 mmHg, and if the RR to maintain this second target was higher than 29, a range of 55–65 mmHg was considered (Table 1).

Monitoring during general anesthesia included peripheral hemoglobin oxygen saturation (SpO_2_%) assessed with a pulse oximetry probe on the tongue; heart rate (HR; beats/minute) obtained from lead II electrocardiography; noninvasive arterial blood pressure (systolic, diastolic, and mean arterial blood pressure: SAP, DAP, and MAP, respectively; mmHg) using an oscillometric method; core body temperature (T; °C) monitored through an esophageal probe; and respiratory rate (RR), EtCO_2_, and FiO_2_ using a multiparametric monitor.

Post-surgery, isoflurane administration was stopped, and dogs were gradually transitioned from mechanical ventilation to spontaneous breathing: the respiratory rate (RR) was gradually reduced by 3 breaths per minute, waiting for a period of two minutes between each step, until a voluntary respiratory effort was observed on the capnographic curve, signaling the transition from mechanical to spontaneous ventilation. Extubation occurred upon restoring the swallowing reflex, with all patients breathing room air between the conclusion of anesthesia and extubation. After extubation, a pediatric helmet (Dimar Air, DIMAR, Modena, Italy) was promptly placed on all patients, receiving oxygen at a flow rate ranging from 6 to 10 L/min through a Venturi Valve, resulting in a measured FiO_2_ inside the helmet of 0.3–0.4 (oxygen–air mixture). In this phase, according to randomization, patients were allocated to receive (YES/NOT) 5 cmH_2_O of CPAP.

### 2.3. Study Protocol

Five minutes after disconnection from the anesthetic circuit, with the endotracheal tube still in place (T_0_) and breathing room air (i.e., FiO_2_ = 0.21), a first arterial blood gas analysis was performed in all patients. At the same time, HR, RR, and MAP were also recorded. Immediately after extubation, patients were allocated, according to a simple randomization scheme, into the CPAP and NO-CPAP groups. In the CPAP group, the PEEP valve of the helmet was adjusted to ensure 5 cmH_2_O of CPAP, with continuous monitoring of CPAP levels using a manometer equipped with the helmet. The PEEP valve was removed from the helmets of dogs in the NO-CPAP group. Helmet tolerance (HT) was assessed in all subjects using a predefined score [14]: 1 = The patient is comfortable; no agitation; no attempts to remove the helmet. 2 = The patient tolerates the helmet but looks stressed and afraid; no attempts to remove the helmet. 3 = The patient tries to remove the helmet and is agitated. It is still possible to keep the helmet on by gently restraining the patient. 4 = The patient does not tolerate the helmet, is restless, attempts to move and put the helmet, and needs extra sedation to tolerate the helmet.

If dogs exhibited an HT score of 4, the helmet was removed, the dog was excluded from the trial, and oxygenation continued with a facemask if necessary. The incidence of helmet intolerance was recorded and analyzed. Heart rate, RR, MAP, CPAP, FiO_2_, and HT were recorded every 10 min during the trial. Peripheral SpO_2_ measurement, assessed with a pulse oximetry probe at an appropriate location, was consistently maintained in all cases during this study, and in case SpO_2_ was lower than 85%, the patient was intubated and supported with ventilation based on the clinician’s assessment of the patient’s condition. In such cases, the trial was interrupted. The incidence of reintubation was recorded and analyzed. Thirty minutes after helmet application (T_30_), a second arterial blood gas analysis was performed for all patients, and HR, RR, HT, and MAP were also recorded. Cases in which obtaining a second arterial blood sample was not feasible were excluded from subsequent analyses.

### 2.4. Gas Blood Assessment

Arterial blood gases were corrected for the patient’s body temperature at the time of sampling. The analyzer provided the SaO_2_ value. PaO_2_/FiO_2_ was calculated as an index to describe pulmonary arterial blood oxygenation.

The alveolar–arterial oxygen gradient of O_2_ [P(A-a) O_2_] was calculated using the alveolar gas equation

P(A-a)O_2_ = [(PB-PH_2_O) × FiO_2_ − PaCO_2_/R] − PaO_2_,

where PB is the barometric pressure, PH_2_O is the water vapor pressure, and R is the respiratory exchange ratio, assumed to be 0.8 [14]. The analyzer measured the PB during each analysis, and the PH_2_O was also corrected for the patient’s rectal temperature.

The estimated percentage of intrapulmonary shunt (F-Shunt) was calculated as follows [19]:

F-Shunt = ([Cc′O_2_ − CaO_2_]/[Cc′O_2_ − CaO_2_ + 3.5 mL dL^−1^]) × 100

where Cc′O_2_ is the pulmonary end-capillary oxygen content, CaO_2_ is the arterial oxygen content, and 3.5 mL dL^−1^ is an approximately fixed value of the arterial-to-mixed venous oxygen content difference. Cc′O_2_ and CaO_2_ were calculated as follows:

Cc′O_2_ = Hb × 1.31 × Sc′O_2_ + 0.0031 × Pc′O_2_

CaO_2_ = Hb × 1.31 × SaO_2_ + 0.003 × PaO_2_

where Hb is the hemoglobin concentration (g dL^−1^), 1.31 is the oxygen-carrying capacity of hemoglobin (mL g^−1^), Sc′O_2_ is the pulmonary end-capillary oxygen saturation, 0.003 is the solubility coefficient of oxygen in dog plasma [20], and Pc′O_2_ is the pulmonary end-capillary partial pressure of oxygen. Pc′O_2_ was assumed to be equal to PAO_2_. For PAO_2_ > 100 mmHg, Pc′O_2_ was assumed to be 100%; for PAO_2_ ≤ 100 mmHg, Pc′O_2_ was calculated from the actual PAO_2_ via the same method.

### 2.5. Statistical Analysis

The sample size was calculated based on previous data [14] and an estimated clinically significant variation in PaO_2_/FiO_2_ of 20%. Power calculation was conducted for a two-tailed *t*-test with a power of 0.8 and an alpha error of 0.05 (Granmo, Version 7.12). The results of this analysis suggested that a minimum of 14 dogs per group would be sufficient to detect significant differences among groups.

All data were analyzed using MedCalc 12.7.0.0 software.

The normal distribution of the data was evaluated using the Shapiro–Wilk test. For all data, the mean and standard deviation were calculated. Data regarding age, bodyweight, anesthesia duration, blood gas assessment at PREM (PaO_2_, PaCO_2_, and pH), RR, HR, and MAP were compared between groups with the one-way ANOVA test, and post hoc analysis was performed with the Tukey test. The postoperative values at T_0_ and at T_30_ of HR, MAP, RR, HT, PaO_2_, PaO_2_/FiO_2_, P(A-a)O_2_, F-Shunt, SaO_2_, PaCO_2_, and helmet tolerance score were analyzed by two-way ANOVA; treatment, time, and treatment-by-time interaction were included in the model as the fixed effects. The Bonferroni test was used for post hoc analysis. A *p* value < 0.05 was considered statistically significant.

## 3. Results

**Figure 1:**
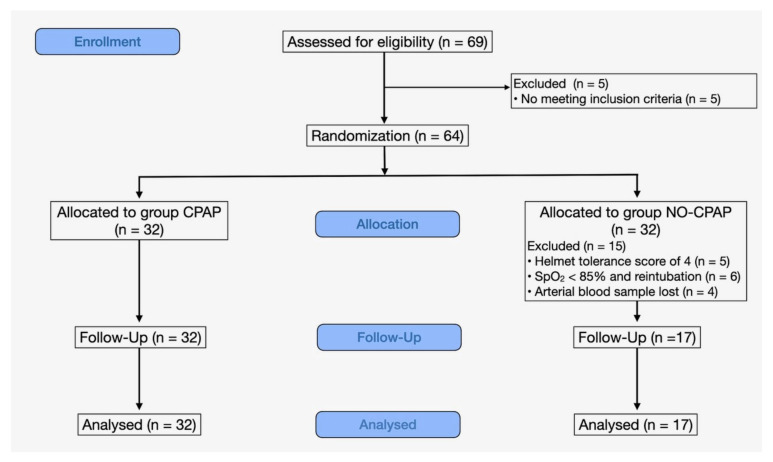
CONSORT flow diagram of the brachycephalic dogs recovering from general anesthesia and receiving noninvasive oxygen support by means of a helmet with (CPAP group) or without (NO-CPAP group) 5 cmH_2_O of CPAP.

A cohort of 69 brachycephalic dogs undergoing surgical procedures was initially assessed for eligibility. Subsequently, five cases were excluded due to non-compliance with inclusion criteria, specifically related to the duration of anesthesia. Consequently, 64 dogs were enrolled, with an equal distribution of 32 in each group. A total of 15 dogs in the NO-CPAP group were excluded from this study based on the exclusion criteria; five were excluded due to an HT score of 4 (5/32, 15.6%), six because of SpO_2_ < 85% and because they were reintubated (6/32, 18%), and four because the second arterial blood sample was not feasible. Therefore, this study was completed by 32 animals in the CPAP group and 17 animals in the NO-CPAP group (Figure 1).

The baseline characteristics of the subjects by intervention group are presented in Table 2, and no statistical differences were observed.

**Figure 2:**
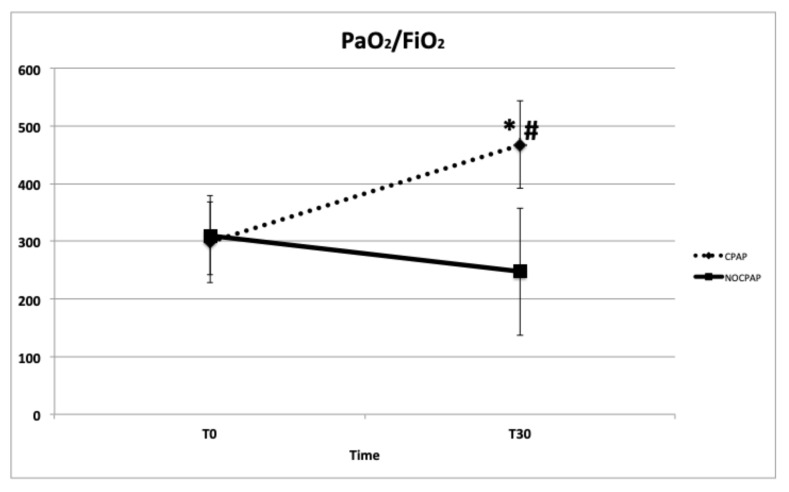
Means ± standard deviations of the ratio of arterial partial oxygen tension to inspired oxygen fraction (PaO_2_/FiO_2_) in brachycephalic dogs recovering from general anesthesia and receiving noninvasive oxygen support by means of a helmet with (CPAP group) or without (NO-CPAP group) 5 cmH_2_O of CPAP. Data are reported at T_0_ immediately prior to support (FiO_2_ = 0.21) and at T_30_ after 30 min of support (FiO_2_ = 0.35–0.40). * *p* < 0.05 within groups and # *p* < 0.05 between groups at T_30_.

**Figure 3:**
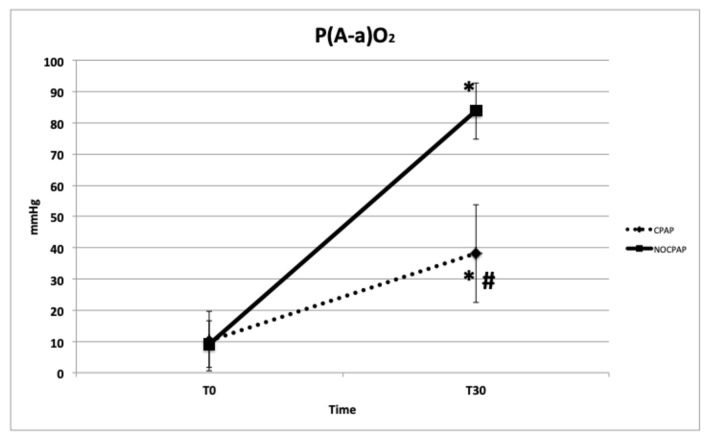
Mean and standard deviation of alveolar–arterial difference of oxygen tension (P(A-a)O_2_) in brachycephalic dogs recovering from general anesthesia and receiving noninvasive oxygen support by means of a helmet with (CPAP group) or without (NO-CPAP group) 5 cmH_2_O of CPAP. Data are reported at T_0_ immediately prior to support (FiO_2_ = 0.21) and at T_30_ after 30 min of support (FiO_2_ = 0.35–0.40). * *p* < 0.05 within groups and # *p* < 0.05 between groups at T_30_.

**Figure 4:**
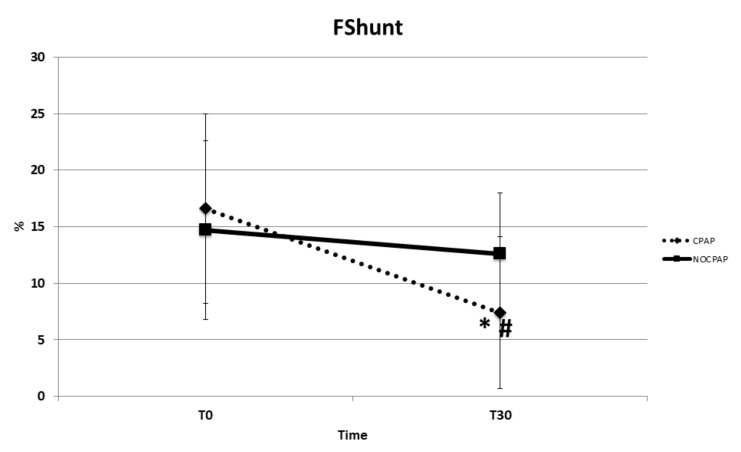
Mean and standard deviation values of estimated intrapulmonary shunt fraction (F-Shunt) in brachycephalic dogs recovering from general anesthesia and receiving noninvasive oxygen support by means of a helmet with (CPAP group) or without (NO-CPAP group) 5 cmH_2_O of CPAP. Data are reported at T_0_ immediately prior to support (FiO_2_ = 0.21) and at T_30_ after 30 min of support (FiO_2_ = 0.35–0.40). * *p* < 0.05 within groups and # *p* < 0.05 between groups at T_30_.

**Figure 5:**
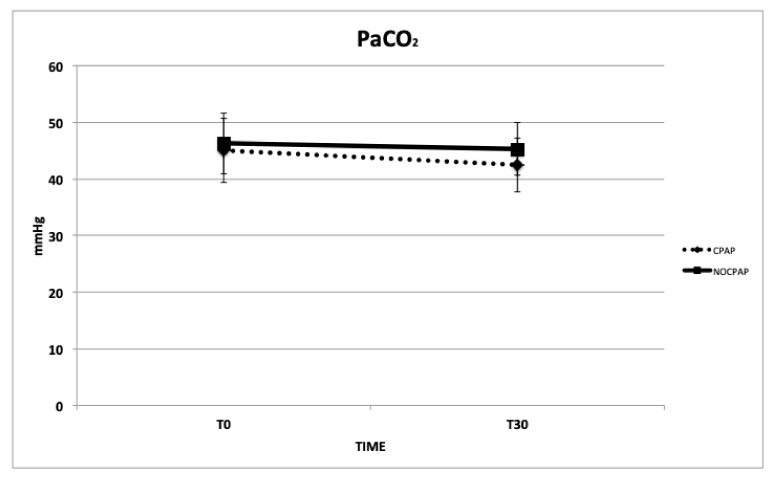
Mean and standard deviation values of arterial partial pressure of carbon dioxide (PaCO_2_) in brachycephalic dogs recovering from general anesthesia and receiving noninvasive oxygen support by means of a helmet with (CPAP group) or without (NO-CPAP group) 5 cmH_2_O of CPAP. Data are reported at T_0_ immediately prior to support (FIO_2_ = 0.21) and at T_30_ after 30 min of support (FIO_2_ = 0.35–0.40).

The mean time between T_0_ and extubation was similar between the two groups: CPAP = 2.1 ± 0.5 min. and NO-CPCP = 1.9 ± 0.4 min. Regarding the primary outcomes, a significant difference was observed between CPAP and NO-CPAP groups regarding PaO_2_ (*p* < 0.001), PaO_2_/FiO_2_ (*p* < 0.001), P(A-a)O_2_ (*p* < 0.001), F-Shunt (*p* < 0.001), and RR (*p* = 0.03). There were no significant differences between the groups in PaCO_2_ (*p* = 0.13), pH (*p* = 0.09), HR and MAP, SaO_2_, and the HT (Table 3 and Figure 2, Figure 3, Figure 4 and Figure 5) between the dogs that completed this study. The incidence of reintubation (18% in the NO-CPAP group vs. 0% in the CPAP group) and HT (15.6% in the NO-CPAP group vs. 0% in the CPAP group) was higher in the NO-CPAP group compared with the CPAP group.

## 4. Discussion

This prospective, multicenter, randomized clinical study demonstrated that applying CPAP therapy using a pediatric helmet with 5 cmH_2_O for 30 min during the recovery phase from general anesthesia in brachycephalic dogs with BOAS enhanced blood oxygenation. Furthermore, compared with a non-CPAP approach, patients receiving 5 cmH_2_O CPAP exhibited reduced complications and a notable helmet tolerance, reflecting a more comfortable recovery.

BOAS is notably prevalent among brachycephalic dogs, a group anatomically predisposed to postoperative hypoxia due to their airway morphology. Consequently, peri-anesthetic mortality rates in brachycephalic breeds are considerably higher than those in non-brachycephalic breeds. Most deaths in brachycephalic breeds occur during the postoperative period mainly due to respiratory complications [21]. Therefore, postoperative airway management in these breeds is paramount, aiming to mitigate the natural tendency for airway obstruction [4].

Helmet CPAP has demonstrated more tolerability in humans than other interfaces used in noninvasive ventilation strategies, offering effective sealing and airway stability. Studies have indicated that, when compared with conventional oxygen therapy, helmet CPAP reduces the intubation rate and lowers mortality [22].

The current study aligns with this pattern. Dogs receiving CPAP with the helmet exhibited a notable tolerance to the interface, contrasting with animals in the NO-CPAP group, which showed intolerance to the helmet and need for reintubation in 15.6% and 18% of the cases, respectively.

Given the specific conditions of this study, we can assume that CPAP administration was the discriminating factor that improved tolerance and reduced the need for reintubation. The only difference between the groups in this study was the CPAP therapy. Data from this study reveal that pre-extubation breathing of room air through the endotracheal tube led to consistently good and comparable oxygenation status between the two groups, suggesting a similar baseline oxygenation level at the end of anesthesia. More precisely, at baseline (T_0_) in both groups, PaO_2_ measured approximately 90 mmHg, the PaO_2_/FiO_2_ ratio hovered around 300, and F-Shunt was approximately 15%. Additionally, the RR and PaCO_2_ levels were similar between groups, suggesting comparable ventilation conditions at the initial measurement.

However, after extubation, airway obstruction activated, and oxygenation deteriorated in the NO-CPAP group, where a standard oxygen supplementation failed to maintain blood oxygenation. Data at T_30_ indicate a significant difference between the two noninvasive ventilatory support techniques used in this study. All in all, helmet CPAP increased PaO_2_ by 78% (91.8 vs. 164 mmHg) and PaO_2_/FiO_2_ by 56.7% (298 vs. 467). In contrast, animals in the NO-CPAP group showed a lower level of oxygenation at T_30_ compared with T_0_ despite the standard oxygen therapy. In the NO-CPAP group, there was a marginal and statistically non-significant increase of 14% in PaO_2_ (96 vs. 110 mmHg), accompanied by a 20% decrease in PaO_2_/FiO_2_ (310 vs. 246). Assuming an average FiO_2_ value of 0.35 and 21% at T_30_ and T_0_, respectively, it can be inferred that using CPAP, known to induce airway distension [17], facilitates improved tidal volume diffusion. These changes, in turn, enhance alveolar ventilation, improving oxygenation [14]. However, we should note that traditional oxygen therapy was sufficient to ensure adequate oxygenation in the NO-CPAP group as well.

This study also revealed that, despite similar PaCO_2_ levels in the CPAP and NO-CPAP groups at T_30_, there was a significant reduction in intrapulmonary shunts and a conserved respiratory rate in the CPAP group. Specifically, data on the intrapulmonary shunt, estimated using the F-Shunt formula, demonstrated that in subjects receiving CPAP, the percentage of shunt decreased by 50% at the end of treatment (16.6% vs. 7.4%) compared with the NO-CPAP group, where F-Shunt remained unchanged (14.7% vs. 12.6%). This result suggests that the increased airway distension provided by the helmet CPAP reduced the work of breathing (decreased resistances), increased lung parenchyma distension, promoted alveolar recruitment, and, subsequently, reduced the intrapulmonary shunt [14,17]. This finding coincides with those reported in dogs with hypoxemic acute respiratory failure in which helmet CPAP allows FiO_2_ to be increased approximately to 40% and significantly decreased F-Shunt by 37% in most dogs [23].

Additional noteworthy data from this study include the reduction in P(A-a)O_2_ observed in the CPAP group at T_30_ compared with the NO-CPAP group. The significant increase in P(A-a)O_2_ in the NO-CPAP group suggests a degree of pulmonary atelectasis, providing insight into the differences between the groups. These findings align with those reported for dogs experiencing hypoxemic acute respiratory failure, where helmet CPAP increased PaO_2_ and the PaO_2_/FiO_2_ ratio, accompanied by a significant decrease in P(A-a)O_2_ after CPAP therapy (pre-CPAP = 52.4 ± 17.07 mmHg vs. post-CPAP = 35.15 ± 22.98 mmHg). These changes suggest potential pulmonary recruitment of collapsed areas [23].

The PaCO_2_ values in dogs in the NO-CPAP group can be attributed to a dramatic 94% increase in RR (19 vs. 27 breaths/min), suggesting a reduction in tidal volume at T_30_ in this population. Although not measured, the hypothetical reduction in tidal volume can be related to airway obstruction leading to postoperative dyspnea. In contrast, these findings were different in the CPAP group, where RR remained unchanged between T_30_ and T_0_, presumably due to ensuring airway patency [17,24,25].

In dogs with BOAS, airway protection through intubation resolves active airway obstruction. However, upon extubation, without support, the work of breathing increases, leading to respiratory fatigue, a gradual reduction in tidal volume, and the need to increase RR to maintain PaCO_2_ levels [4]. This condition usually persists until the complete recovery of the case. Considering the anesthesia protocol used in this study and the type of cases, we have limited our observation to 60 min, but it may require longer time in some specific cases.

Although there is no evidence in the veterinary or human literature of adverse effects of postoperative hemodynamic effects of CPAP, we can assume a potential interference of increased intrathoracic pressure on venous return [26]. Our data demonstrated that MAP was not affected by CPAP, as it remained in a physiological range at the study times in the specific population of dogs included in this study.

Helmet CPAP, as an alternative to noninvasive ventilatory support, appears promising for enhancing ventilation and blood oxygenation during anesthetic recovery in brachycephalic breeds. The level of CPAP administered was arbitrarily chosen based on the literature; however, it can be adapted to the needs of the case. Further studies are warranted to confirm this observation.

High-flow nasal cannula (HFNC) is an alternative technique for noninvasive respiratory support that has recently been introduced in veterinary medicine [27]. It involves administering a very high (20–60 L/min), humidified, and heated gas flow into the animal’s nostrils [27]. This technique has been evaluated in a few cases for postoperative respiratory support in brachycephalic dogs [11]. The authors were able to prove that HFNC reduced dyspnea over time. However, this study did not have a control group and lacked any oxygenation assessment. For this reason, this study cannot be compared with our results, and future researchers can compare the two noninvasive respiratory support techniques in brachycephalic breeds. Additional studies were also recently published showing the potential positive effects of HFNC in hypoxemic dogs [28,29,30].

This study has several limitations. Firstly, we did not stratify our population of dogs and results to consider the severity of BAOS, an aspect future studies may find valuable to explore. Additionally, the potential bias introduced by the unequal completion rates in the two groups can impact the results; however, this study’s minimum required number of cases for power calculation was maintained. Lean body weight was not considered to set the tidal volume during anesthesia, and this may have created some bias in the results.

Moreover, our observation was confined to the initial 30 min of the postoperative period, and extending the observation period might have yielded additional insightful information. Future studies can address these limitations to enhance the comprehensiveness and robustness of the findings.

## 5. Conclusions

In conclusion, this study underscores the importance of strategic perioperative management in brachycephalic breeds, with a specific focus on the recovery phase. The application of CPAP, particularly via a pediatric helmet, demonstrates promising results in improving oxygenation during the recovery period. Further investigations and larger-scale studies are warranted to validate and generalize these findings, ultimately contributing to enhanced care for brachycephalic dogs undergoing surgical procedures.

## Figures and Tables

**Table 1:** Predetermined stepwise protocol for adjusting respiratory rate (RR) to end-tidal CO_2_ (EtCO_2_) during volume-control mode ventilation in brachycephalic dogs undergoing surgical procedures exceeding 60 min.

RR (Breaths/Minute)	EtCO_2_ (mmHg)
<20	40–45
<30	45–55
<40	55–65

**Table 2:** Number of animals within each group that completed this study and respective data (mean ± standard deviation) of sex, age, bodyweight, duration of anesthesia, respiratory rate (RR), heart rate (HR), mean arterial pressure (MAP), arterial oxygen (PaO_2_) and carbon dioxide (PaCO_2_) tensions, and pH at premedication (PREM).

	CPAP	NO-CPAP	*p*
Dogs (*n*)	32	17	0.34
Sex (female/male; *n*)	18/14	11/6	0.67
Age (months)	4.3 ± 3.0	4.9 ± 3.4	0.13
Bodyweight (kg)	15.8 ± 7.5	15.6 ± 5.4	0.21
Duration of anesthesia (minutes)	82 ± 13	88 ± 12	0.12
RR (breaths/minute) at PREM	27 ± 15	18 ± 5	0.11
HR (beats/minutes) at PREM	103 ± 18	107 ± 28	0.23
MAP (mmHg) at PREM	84 ± 5	84 ± 5	0.31
PaO_2_ (mmHg)	95 ± 6	93 ± 4	0.21
PaCO_2_ (mmHg)	34.1 ± 2.4	36.5 ± 3.6	0.09
pH	7.39 ± 0.02	7.34 ± 0.12	0.12

**Table 3:** Mean ± standard deviation of respiratory rate (RR), heart rate (HR), mean arterial blood pressure (MAP), arterial oxygen (PaO_2_) and carbon dioxide (PaCO_2_) tensions, the PaO_2_ to inspired oxygen tension ratio (PaO_2_/FiO_2_), the estimated intrapulmonary shunt (F-Shunt), the arterial O_2_ saturation (SaO_2_), and the helmet tolerance score (HF) in the postoperative period of dogs that completed this study divided in the two groups with (CPAP) and without (NO-CPAP) 5 cmH_2_O of CPAP. * *p* < 0.05 within groups and # *p* < 0.05 between groups at T_30_.

Parameters	Group	T_0_	T_30_
RR (breaths/min)	CPAP NO-CPAP	19.6 ± 9.1 19.1± 4.8	19.8 ± 10.5 # 27.7 ± 12.6
HR	CPAP NO-CPAP	104 ± 24 99 ± 15	98 ± 18 93 ± 13
MAP	CPAP NO-CPAP	82 ± 7 81± 7	82 ± 10 84 ± 9
PaO_2_ (mmHg)	CPAP NO-CPAP	91.8 ± 20.4 96.1 ± 14.3	164.7 ± 27.5 *# 110.4 ± 17.3
PaO_2_/FiO_2_	CPAP NO-CPAP	298.1 ± 69.6 310.5 ± 68.3	467.1 ± 75.9 *# 246.9 ± 109.8 *
P(A-a)O_2_ mmHg	CPAP NO-CPAP	10.2 ± 9.6 9.1 ± 7.5	38.1 ± 15.6 *# 83.8 ± 9.1 *
F-Shunt (%)	CPAP NO-CPAP	16.6 ± 8.4 14.7 ± 7.9	7.4 ± 6.7 *# 12.6 ± 5.4
SaO_2_	CPAP NO-CPAP	95.4 ± 3.1 95.2 ± 2.1	98.8 ± 1.5 98.2 ± 1.5
PaCO_2_ (mmHg)	CPAP NO-CPAP	45.1 ± 5.7 46.3 ± 5.4	42.5 ± 4.8 45.3 ± 4.7
HT	CPAP NO-CPAP	2.4 ± 0.7 3.2 ± 0.5	1.8 ± 1.1 2.9 ± 0.7

* *p* < 0.05 within groups; # *p* < 0.05 between groups at T_30_.

## Data Availability

Data is contained within the article.
